# Association of Brain Reward Learning Response With Harm Avoidance, Weight Gain, and Hypothalamic Effective Connectivity in Adolescent Anorexia Nervosa

**DOI:** 10.1001/jamapsychiatry.2018.2151

**Published:** 2018-07-19

**Authors:** Guido K. W. Frank, Marisa C. DeGuzman, Megan E. Shott, Mark L. Laudenslager, Brogan Rossi, Tamara Pryor

**Affiliations:** 1 Department of Psychiatry, University of Colorado Anschutz Medical Campus, School of Medicine, Aurora; 2 Neuroscience Program, University of Colorado Anschutz Medical Campus, Aurora; 3 Eating Disorder Care, Denver, Colorado

## Abstract

**Importance:**

Anorexia nervosa (AN) is associated with adolescent onset, severe low body weight, and high mortality as well as high harm avoidance. The brain reward system could have an important role in the perplexing drive for thinness and food avoidance in AN.

**Objective:**

To test whether brain reward learning response to taste in adolescent AN is altered and associated with treatment response, striatal-hypothalamic connectivity, and elevated harm avoidance.

**Design, Setting, and Participants:**

In this cross-sectional multimodal brain imaging study, adolescents and young adults with AN were matched with healthy controls at a university brain imaging facility and eating disorder treatment program. During a sucrose taste classical conditioning paradigm, violations of learned associations between conditioned visual and unconditioned taste stimuli evoked the dopamine-related prediction error (PE). Dynamic effective connectivity during sweet taste receipt was studied to investigate hierarchical brain activation across the brain network that regulates eating. The study was conducted from July 2012 to May 2017, and data were analyzed from June 2017 to December 2017.

**Main Outcomes and Measures:**

Prediction error brain reward response across the insula, caudate, and orbitofrontal cortex; dynamic effective connectivity between hypothalamus and ventral striatum; and treatment weight gain, harm avoidance scores, and salivary cortisol levels and their correlations with PE brain response.

**Results:**

Of 56 female participants with AN included in the study, the mean (SD) age was 16.6 (2.5) years, and the mean (SD) body mass index (BMI; calculated as weight in kilograms divided by height in meters squared) was 15.9 (0.9); of 52 matched female controls, the mean (SD) age was 16.0 (2.8) years, and the mean (SD) BMI was 20.9 (2.1). Prediction error response was elevated in participants with AN in the caudate head, nucleus accumbens, and insula (multivariate analysis of covariance: Wilks λ, 0.707; *P* = .02; partial η^2^ = 0.296), which correlated negatively with sucrose taste pleasantness. Bilateral AN orbitofrontal gyrus rectus PE response was positively correlated with harm avoidance (right ρ, 0.317; 95% CI, 0.091 to 0.539; *P* < .02; left ρ, 0.336; 95% CI, 0.112 to 0.550; *P* < .01) but negatively correlated with treatment BMI change (right ρ, −0.282; 95% CI, −0.534 to −0.014; *P* < .04; left ρ, −0.268; 95% CI, −0.509 to −0.018; *P* < .045). Participants with AN showed effective connectivity from ventral striatum to hypothalamus, and connectivity strength was positively correlated with insula and orbitofrontal PE response. Right frontal cortex PE response was associated with cortisol, which correlated with body dissatisfaction.

**Conclusions and Relevance:**

These results further support elevated PE signal in AN and suggest a link between PE and elevated harm avoidance, brain connectivity, and weight gain in AN. Prediction error may have a central role in adolescent AN in driving anxiety and ventral striatal-hypothalamus circuit-controlled food avoidance.

## Key Points

**Question:**

How does brain response in participants with adolescent anorexia nervosa (AN) compare with healthy controls during taste reward conditioning?

**Findings:**

In this cross-sectional multimodal brain imaging study of 56 female adolescents and young adults with AN and 52 matched controls, the AN group showed hyperactivation in the caudate head, nucleus accumbens, and insula compared with controls during a classical conditioning paradigm that has been associated with dopamine function. Orbitofrontal brain response in the AN group was positively associated with harm avoidance and striatal-hypothalamic connectivity but negatively associated with change in body mass index during treatment.

**Meanings:**

Altered brain reward response in adolescent AN may indicate altered dopamine function, may have a key role in AN’s specific pathophysiology, and should be explored as a target for biological treatments.

## Teaser

This cross-sectional multimodal brain imaging study examines whether brain reward learning response to taste in adolescent anorexia nervosa is altered and associated with treatment response, striatal-hypothalamic connectivity, and elevated harm avoidance.

## Introduction

Anorexia nervosa (AN) is a psychiatric disorder characterized by fear of weight gain and dangerously low body weight.^1^ Anorexia nervosa primarily affects young girls and young women, and its mortality rate exceeds that of other psychiatric disorders.^2^ Self-starvation and fear of weight gain despite severe underweight and risk of death have been puzzling, and finding comprehensive brain-based models that could explain the constellation of these behaviors has been difficult.^3^

It has been hypothesized that anxious traits are vulnerability factors for AN; specifically, elevated harm avoidance (HA) has been found in individuals with AN.^4,5,6^ Harm avoidance is a temperament trait characterized by excessive worry and fearfulness that has been associated with poor AN treatment outcome and is higher in the ill state compared with the recovered state.^7,8^ Harm avoidance was found to be correlated with serotonin neurotransmitter receptor availability and, more recently, dopamine receptor binding in AN after recovery.^3,9,10,11,12,13^ Dopamine is a learning signal and is important for food approach, and animal models suggest enhanced neuronal dopamine activation following food restriction.^14,15^ This led to the hypothesis that brain circuits that involve dopamine are important for the pathophysiology of AN.^16,17^ Unexpected reward receipt and omission have been associated with brain dopamine level, the so-called prediction error (PE) response, and have been studied in AN.^18,19^ In adults with AN in the ill and recovered states, unexpected or randomly applied sucrose taste stimuli evoked higher insular and striatal responses,^20,21^ and unexpected omission or receipt of monetary reward in adolescent AN also resulted in heightened responses in those regions.^22^ One interpretation of such findings may lend itself to a model of brain changes in AN: an enhanced dopamine reward system response is an adaptation to starvation to stimulate motivation to approach food.^14^ Notably, peak onset of AN is in midadolescence,^23^ when sensitivity to reward and reward PE response is still developing.^24,25,26^ Individuals vulnerable to develop AN could be particularly sensitive to food restriction and adaptations of reward response during that developmental period.

In this study, we tested specific hypotheses to integrate PE reward signals with anxiety and core eating disorder signs.^17^ Specifically, we hypothesized that reward learning PE response would be elevated in AN and associated with HA. Second, we wanted to test the hypothesis that PE response is associated with neurocircuitry that regulates appetite and food intake. Previously, we found a pattern of effective connectivity (direction of activation) from ventral striatum to hypothalamus in adults with AN.^27^ We interpreted this as a possible mechanism of how the brain could override hunger signals in AN. We hypothesized that adolescents with AN show a similar pattern. Third, food restriction is a stressor and is associated with increased brain cortisol levels.^28,29,30^ Cortisol level affects dopamine release and postsynaptic dopamine D2 receptors,^31,32,33^ and we wanted to test whether cortisol level was associated with PE response and core AN behaviors.^34,35,36^

## Methods

### Participants

Fifty-six female adolescents and young adults with AN (age range, 11-21 years) and 52 healthy matched control participants (age range, 11-21 years) were included in this study (Table 1).^37,38,39,40,41^ The AN group was recruited from partial hospitalization treatment, where closely supervised meal plans mitigated confounding brain effects of acute starvation or dehydration.^42^ Treatment involved a highly structured program aimed at weight restoration over 5 weeks, including parent training in meal support according to the family-based treatment model.^43^ Control participants were recruited through local advertisements. In the AN group, 53 participants were diagnosed as having pure restricting type and 3 as having infrequent purge episodes (less than once a month), and all 56 participants with AN fell below the 10th percentile for body mass index (BMI; calculated as weight in kilograms divided by height in meters squared) for age. All participants underwent functional magnetic resonance imaging (fMRI); individuals with AN were without menses and controls were in the early follicular phase to control for sex hormone effects. Participants 18 years or older, including 19 participants with AN and 11 controls, were administered the Structured Clinical Interview for *DSM*-*5* by a doctoral-level interviewer. Those younger than 18 years completed the Mini-International Neuropsychiatric Interview.^44^ Participants were right-handed and had no history of head trauma, neurological disease, major medical illness, psychosis, or substance use disorders. Six participants with AN and 11 controls were taking oral contraceptives. Twenty-six participants with AN were taking antidepressants, and 7 were taking atypical antipsychotics. The Colorado Multiple institutional review board approved the study. All participants provided written informed consent.

**Table 1.** Demographic and Behavioral Variables

Variable	Mean (SD)		*t*	*P* Value
	Anorexia Nervosa Group (n = 56)	Control Group (n = 52)		
Age, y	16.56 (2.47)	16.01 (2.80)	−1.078	.28
BMI^a^	15.88 (0.86)	20.86 (2.07)	16.125	<.001
Age-adjusted BMI percentile	2.36 (2.63)	58.57 (21.94)	17.081	<.001
Drive for thinness score^b^	19.38 (7.13)	2.13 (3.05)	−16.422	<.001
Body dissatisfaction score^b^	24.76 (10.20)	3.62 (4.32)	−14.103	<.001
Punishment sensitivity score^c^	12.57 (4.02)	5.54 (3.66)	−9.481	<.001
Reward sensitivity score^c^	7.25 (3.83)	6.65 (3.76)	−0.815	.42
State anxiety score^d^	51.89 (13.85)	28.00 (6.29)	−11.590	<.001
Trait anxiety score^d^	53.05 (13.57)	29.48 (7.05)	−11.440	<.001
Harm avoidance score^e^	21.98 (7.43)	10.79 (4.80)	−9.362	<.001
Reward dependence score^e^	14.64 (3.58)	15.77 (3.67)	1.616	.11
Depression score^f^	18.16 (9.74)	2.90 (2.85)	−10.008	<.001
Breakfast calories	602.857 (145.42)	568.75 (151.12)	−1.195	.24
Sucrose pleasantness score	4.32 (2.41)	5.08 (2.56)	1.646	.10
Sucrose sweetness score	8.00 (1.24)	8.06 (1.07)	0.258	.80
Antidepressant use, No. (%)	26 (46.4)	NA	NA	NA
Antipsychotic use, No. (%)	7 (12.5)	NA	NA	NA
Mood disorder, No. (%)	20 (35.7)	NA	NA	NA
Anxiety disorder, No. (%)	28 (50.0)	NA	NA	NA

Abbreviations: BMI, body mass index; NA, not applicable.

^a^ Calculated as weight in kilograms divided by height in meters squared.

^b^ Eating Disorder Inventory–3.^37^

^c^ Revised Sensitivity to Punishment and Reward Questionnaire.^38^

^d^ State-Trait Anxiety Inventory.^39^

^e^ Temperament and Character Inventory.^40^

^f^ Children’s Depression Inventory.^41^

### Self-Assessments

In addition to diagnostic interviews, participants completed a battery of self-assessments. Participants completed the Eating Disorder Inventory–3,^37^ Revised Sensitivity to Punishment and Reward Questionnaire,^38^ State-Trait Anxiety Inventory,^39^ Temperament and Character Inventory,^40^ and Children’s Depression Inventory.^41^

### Brain Imaging Methods

#### fMRI Image Acquisition

Between 7:00 am and 9:00 am on the study day, participants with AN ate their meal plan breakfast and controls ate a quality-matched and calorie-matched breakfast (Table 1). Brain imaging was performed between 8:00 am and 9:00 am using the 3T Signa scanner (General Electric Company) or Skyra 3T scanner (Siemens) using the following criteria: 3-plane scout scan (16 seconds), sagittally acquired, spoiled gradient sequence T1-weighted (172 slices; thickness, 1 mm; inversion time, 450 ms; repetition time, 8 ms; echo time, 4 ms; flip angle, 12°; field of view, 22 cm; scan matrix, 64 × 64), and T2-weighted echo planar scans for blood oxygen level–dependent functional activity (3.4 × 3.4 × 2.6-mm voxels; repetition time, 2100 ms; echo time, 30 ms; flip angle, 70°; 28 axial slices; thickness, 2.6 mm; gap, 1.4 mm) (eMethods 1 in the Supplement).

#### Taste Reward Task

The design of this study was adapted from O’Doherty et al^19^ (eAppendix 1 in the Supplement). Participants learned to associate 3 unconditioned taste stimuli (1 molar sucrose solution, no solution, or artificial saliva) with paired conditioned visual stimuli. Each conditioned visual stimulus was probabilistically associated with its unconditioned taste stimulus such that 20% of sucrose and no solution conditioned visual stimulus trials were unexpectedly followed by no solution and sucrose unconditioned taste stimuli, respectively. Taste stimuli were applied using a customized programmable syringe pump (J-Kem Scientific) and E-Prime software version 2 (Psychological Software Tools).^45^

#### fMRI Analysis

Image preprocessing and analysis were performed using statistical parametric mapping version 12 (Wellcome Trust Centre for Neuroimaging). Images were realigned to the first volume, normalized to the Montreal Neurological Institute template, and smoothed at 6-mm full width at half maximum gaussian kernel. Data were preprocessed with slice time correction and modeled with a hemodynamic response convolved function using the general linear model, including temporal and dispersion derivatives. A 128-second high-pass filter was applied for low-frequency blood oxygen level–dependent signal fluctuations and motion parameters as first-level analysis regressors.

#### PE Analysis

Each participant’s PE signal was modeled based on trial sequence (absolute of positive and negative PE) and regressed with brain activation across all trials^19,21,22^ (eMethods 2 in the Supplement). We extracted mean parameter estimates across all voxels from 18 predefined anatomical regions of interest (ROIs) based on previous studies,^22^ including the bilateral dorsal anterior insula, ventral anterior insula, caudate head, orbitofrontal cortex (OFC) gyrus rectus, medial OFC, middle OFC, inferior OFC, ventral striatum,^46^ and nucleus accumbens^47^ (http://marsbar.sourceforge.net/; automated anatomical labeling Atlas^48^).

#### Effective Connectivity Analysis

We extracted ROI functional activation for trials of expected receipt of 1 molar sucrose solution (n = 80), with conditioned visual stimuli and unconditioned taste stimuli trial length of 6 seconds, as previously studied in adults.^27^ The Tetrad-V program^49^ was used to infer effective connectivity with independent multisample greedy equivalence search and linear nongaussian orientation, fixed structure search algorithms. This analysis aimed to understand causal associations among neuronal populations whose activity gives rise to observed fMRI signals in spatially localized ROIs^27^ (eMethods 3 in the Supplement). We extracted edge coefficients for ventral striatum–hypothalamus connectivity to test for correlations with behavior or PE values.

### Cortisol Collection and Analysis

On the scan day, a subset of 20 participants with AN and 25 controls provided 0.5-mL samples of saliva (passive drool) in 5-mL Screw Cap Micro Tubes (Thermo Fisher Scientific; eAppendix 2 and eTable 1 in the Supplement). Samples were collected 30 minutes prior to breakfast, 30 minutes after breakfast, and right before brain imaging. Samples were stored at −15**°** C until analysis. Cortisol was assayed using commercial immunoassays (Salimetrics). The area under the curve (AUC; trapezoid method with 3 time points) was calculated and correlated with ROI PE response and across the whole brain (family-wise error rate [FWE] *P* < .05; eAppendix 2 in the Supplement).

### Statistical Analysis

SPSS Statistics 25 (IBM) was used for statistical analyses. Demographic and behavior data were analyzed using *t* test. Extracted regional brain activation parameter estimates were tested for normality with the Shapiro-Wilk test, rank-transformed when nonnormally distributed, and analyzed using multivariate analysis of variance and multivariate analysis of covariance with covariates to account for confounding factors, such as comorbidity or medication, as in previous studies.^22^ Spearman rank was used for correlation analyses and controlled for multiple comparisons using bootstrapping procedures (1000 samples).^50^ All *P* values were 2-tailed, and a *P *value less than .05 was considered significant. Results were corrected for multiple comparison.

## Results

### Demographic and Behavioral Data

There were no significant group differences for age, breakfast calories, or sucrose pleasantness or sweetness ratings (Table 1). Participants with AN had significantly lower age-adjusted BMI percentile and novelty-seeking scores. Participants with AN also had elevated drive for thinness and body dissatisfaction and significantly higher HA, punishment sensitivity, state and trait anxiety, and depression scores.

### Brain Imaging Results

#### PE ROI Analysis

Results were nonnormally distributed. Multivariate analysis of variance (no covariates) resulted in a Wilks λ of 0.642 (*P* < .001; partial η^2^ = 0.358), with associations with bilateral caudate head, ventral striatum, nucleus accumbens, right inferior OFC, right medial OFC, right gyrus rectus, right dorsal anterior insula, and right ventral anterior insula surviving Bonferroni correction (Table 2). Multivariate analysis of covariance (with age, scanner, antidepressant use, antipsychotic use, comorbid depression, and comorbid anxiety as covariates) resulted in a Wilks λ of 0.707 (*P* = .02; partial η^2^ = 0.296), with associations with right and left caudate head, right and left nucleus accumbens, and right ventral anterior insula surviving Bonferroni correction. There were no significant differences between scanners for within-group ROI comparisons, nor did results change when the scanner covariate was removed (eFigure 1 in the Supplement).

**Table 2.** Parameter Estimate Analyses Across Groups^a^

Region of Interest	Response, Mean (SD)		MANOVA			MANCOVA^b^		
	Anorexia Nervosa Group (n = 56)	Control Group (n = 52)	*F*	*P* Value^c^	η_p_^2^	*F*	*P* Value^c^	η_p_^2^
Right caudate head	67.179 (30.158)	40.846 (26.660)	22.972	<.001	0.178	8.102	.005	0.075
Left caudate head	67.786 (30.206)	40.192 (25.917)	25.772	<.001	0.196	13.004	<.001	0.115
Right ventral striatum	57.232 (33.889)	45.134 (27.793)	8.646	.004	0.075	2.904	.09	0.028
Left ventral striatum	62.750 (32.410	45.615 (27.752)	9.695	.002	0.084	3.048	.08	0.030
Right nucleus accumbens	66.196 (30.972)	41.904 (26.678)	18.939	<.001	0.152	8.143	.005	0.075
Left nucleus accumbens	65.857 (30.064)	42.269 (28.094)	17.676	<.001	0.143	4.878	.03	0.047
Right inferior orbitofrontal cortex	61.804 (32.709)	46.635 (27.977)	6.659	.01	0.059	2.925	.09	0.028
Left inferior orbitofrontal cortex	59.161 (32.977)	49.481 (28.911)	2.614	.11	0.024	0.873	.35	0.009
Right medial orbitofrontal cortex	60.411 (33.670)	48.135 (27.494)	4.269	.04	0.039	1.149	.29	0.011
Left medial orbitofrontal cortex	59.804 (32.777)	48.788 (28.904)	3.410	.07	0.031	1.123	.29	0.011
Right middle orbitofrontal cortex	58.268 (32.302)	50.442 (30.009)	1.694	.20	0.016	0.089	.77	0.001
Left middle orbitofrontal cortex	57.661 (32.559)	51.096 (29.869)	1.186	.28	0.011	0.004	.95	0.000
Right gyrus rectus	59.393 (32.503)	49.231 (29.399)	5.953	.02	0.053	0.361	.55	0.004
Left gyrus rectus	57.607 (32.217)	51.154 (30.279)	1.152	.29	0.011	0.011	.92	0.0001
Right dorsal anterior insula	60.339 (31.840)	48.212 (29.784)	4.162	.04	0.038	2.053	.16	0.020
Left dorsal anterior insula	58.732 (32.410)	49.942 (29.742)	2.146	.15	0.020	0.256	.61	0.003
Right ventral anterior insula	62.607 (32.984)	45.769 (27.112)	8.326	.005	0.073	6.773	.01	0.063
Left ventral anterior insula	57.232 (33.889)	51.558 (28.331)	0.884	.35	0.008	0.033	.86	0.0001

Abbreviations: MANCOVA, multivariate analysis of covariance; MANOVA, multivariate analysis of variance.

^a^ Data were rank-transformed.

^b^ Multivariate analysis of covariance included age, scanner, antidepressant use, antipsychotic use, comorbid depression, and comorbid anxiety.

^c^ *P* values are adjusted for Bonferroni multiple comparisons. Rank values remained the same for both analyses.

#### Effective Connectivity

**Figure 1.**
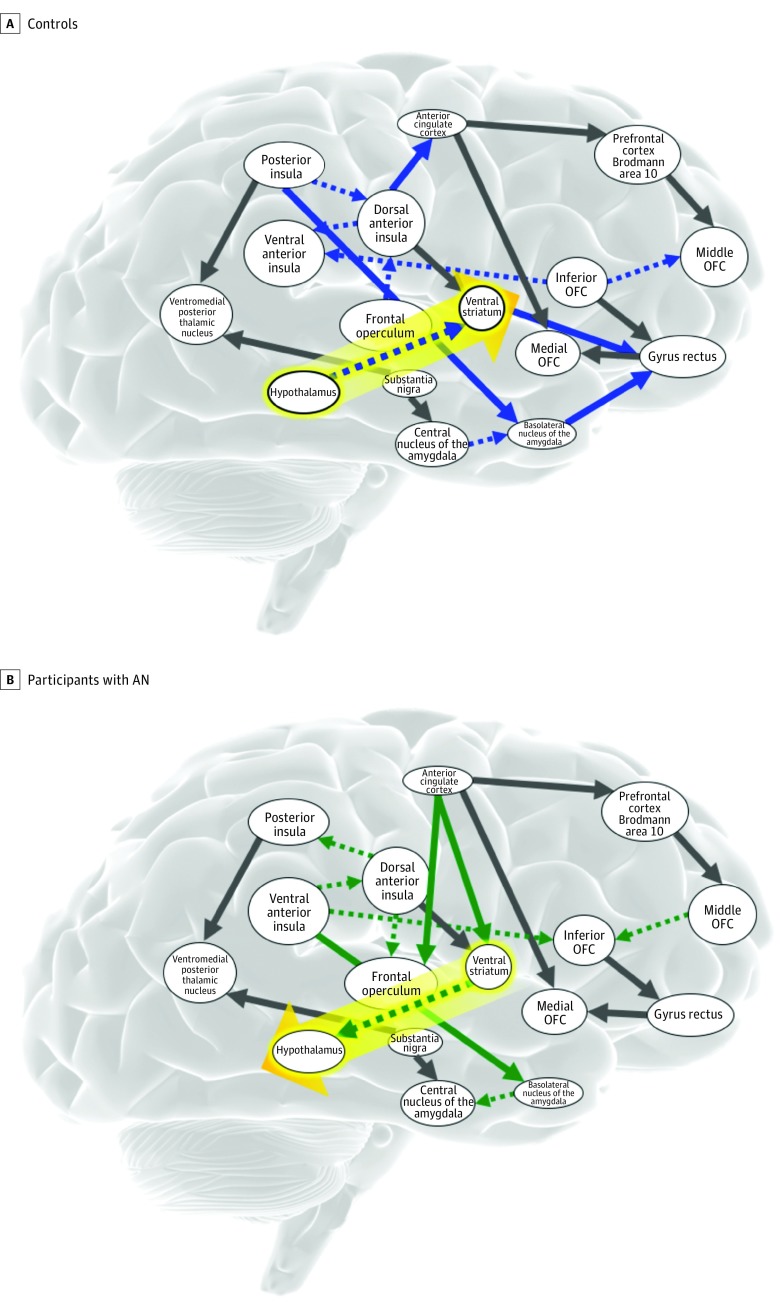
Effective Connectivity of Sucrose Receipt The effective connectivity pattern in healthy controls (A) from the right hypothalamus to the ventral striatum is the opposite pattern seen in participants with anorexia nervosa (AN) (B). The connectivity patterns indicate directionality within group but do not reflect a direct group contrast. The blue arrows indicate patterns unique to controls; the green arrows, patterns unique to participants with AN. OFC indicates orbitofrontal cortex.

Sucrose anticipation and receipt elicited patterns of connectivity that were similar for 50% of identified connections across groups. However, for our effective connectivity of interest, there were different patterns between groups bilaterally; the hypothalamus in controls directed activation to the ventral striatum, whereas in participants with AN, the ventral striatum directed effective connectivity to the hypothalamus (Figure 1).

#### Correlation Analyses

Rate of age-adjusted change of BMI percentile to reach target weight (BMI percentile change mean [SD] of 20.37 [15.12] over a mean [SD] time of 40.01 [10.94] days = 0.51 age-adjusted BMI percentile change per day) was negatively correlated with response in the inferior OFC (right ρ, −0.389; 95% CI, −0.612 to −0.100; *P* < .003), middle OFC (right ρ, −0.281; 95% CI, −0.522 to −0.018; *P* < .04), gyrus rectus (right ρ, −0.282; 95% CI, −0.534 to −0.014; *P* < .04; left ρ, −0.268; 95% CI, −0.509 to −0.018; *P* < .045), dorsal anterior insula (right ρ, −0.358; 95% CI, −0.590 to −0.092; *P* < .007; left ρ, −0.281; 95% CI, −0.542 to −0.004; *P* < .04), and ventral anterior insula (right ρ, −0.274; 95% CI, −0.512 to −0.016; *P* < .04). Prediction error regression weights did not significantly correlate with admission age-adjusted BMI percentiles. In participants with AN, HA was positively correlated with response in OFC gyrus rectus (right ρ, 0.317; 95% CI, 0.091 to 0.539; *P* < .02; left ρ, 0.336; 95% CI, 0.112 to 0.550; *P* < .01). Harm avoidance in participants with AN was positively correlated with drive for thinness (ρ, 0.381; 95% CI, 0.140 to 0.587; *P* < .004) and body dissatisfaction (ρ, 0.312; 95% CI, 0.055 to 0.541; *P* < .02) (eFigure 2 in the Supplement).

**Figure 2.**
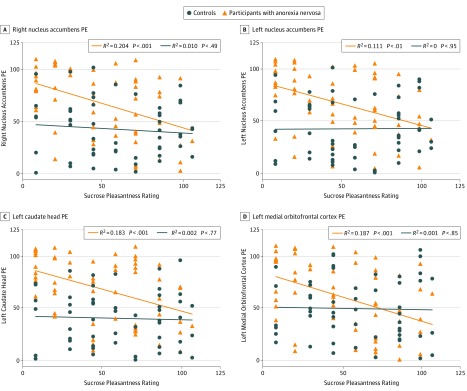
Correlation of Prediction Error (PE) With Taste Pleasantness Regional PE results across groups and their correlation with taste pleasantness ratings for 1 molar sucrose solution. Prediction errors and pleasantness ratings were rank-transformed. See eTable 2 in the Supplement for full correlation results across regions and groups.

Sucrose pleasantness was negatively correlated with PE in all regions studied (ρ range, −0.451 to −0.249) in participants with AN but only in the middle OFC in controls. A comparison of regression slopes showed significantly different slopes between groups in the caudate head (right Fisher *z*, 2.103; *P* < .04), medial OFC (left Fisher *z*, 2.204; *P* < .03), and nucleus accumbens (right Fisher *z*, 1.958; *P* < .050; left Fisher *z*, 2.293; *P* < .02) (Figure 2) (eTable 2 in the Supplement). In the AN group, taste pleasantness was negatively correlated with HA (ρ, −0.294; 95% CI, −0.041 to −0.510; *P* < .03). In participants with AN, ventral striatum–hypothalamus edge coefficients were correlated with ipsilateral inferior OFC PE (right ρ, 0.318; 95% CI, 0.063 to 0.547; *P* < .02; left ρ, 0.354; 95% CI, 0.059 to 0.606; *P* < .007), middle OFC PE (right ρ, 0.308; 95% CI, 0.044 to 0.554; *P* < .02; left ρ, 0.427; 95% CI, 0.142 to 0.646; *P* < .001), and dorsal anterior insula PE (right ρ, 0.392; 95% CI, 0.170 to 0.602; *P* < .003).

#### Cortisol Analysis

The area under the curve for cortisol levels was elevated in participants with AN compared with controls (*t*_29.169_ = −2.515; *P* = .02) (eFigure 3 in the Supplement). In participants with AN, the AUC for cortisol levels was positively correlated with caudate head PE (right ρ, 0.0457; 95% CI, 0.122 to 0.692; *P* < .04). Whole-brain regression (FWE corrected) showed that cortisol level was significantly positively correlated with PE response in the right superior frontal gyrus (x = −18; y = 58; z = 6) in participants with AN (peak FWE *P* = .005; κ = 1). Subsequent small volume correction (*P* < .001; κ = 10) within the anatomical superior frontal gyrus ROI resulted in a significant cluster (peak FWE *P* < .001; κ = 53) (eAppendix 2 in the Supplement). When parameter estimates were extracted from the FWE-corrected cluster, the strength of the cortisol regression was significantly positively correlated with body dissatisfaction scores in participants with AN (ρ, 0.484; 95% CI, 0.013 to 0.815; *P* < .03) (eFigure 3 in the Supplement). No significant clusters or behavioral correlations were found in the control group.

## Discussion

Anorexia nervosa is a perplexing psychiatric illness, and the complex biopsychosocial aspects of the illness have made it difficult to develop brain-based models for food restriction. The results from this study show (1) heightened caudate, nucleus accumbens, and insula taste PE signal in adolescents with AN; (2) ventral striatal–hypothalamic dynamic effective connectivity in participants with AN that was opposite to controls and that was positively correlated with insular and orbitofrontal PE signal; (3) a positive correlation of cortisol level with PE in a subset of participants; and (4) a positive correlation of OFC PE with HA and a negative correlation of orbitofrontal and insula PE with BMI change during treatment, as seen previously.^22^ The results suggest that the PE signal could be an important marker for weight gain and anxiety in adolescent AN.

The first part of this study shows heightened taste reward PE signal across the caudate, nucleus accumbens, and insula in a large sample of adolescents and young adults with AN, comparable with results in adults.^21^ Starvation is associated with adaptations of the body to drive food intake,^51,52,53^ including changes in dopamine release and receptor expression.^53^ We expect that in AN, the elevated PE response, which has been associated with brain dopamine activity, is an adaptation to food restriction and weight loss that normalizes with long-term recovery.^54^

The second major finding was a pattern of activation during sweet taste anticipation and receipt that was directed from the ventral striatum to the hypothalamus in AN and was positively correlated with OFC and insular PE signal. This was in contrast to the direction of activation in controls from the hypothalamus to the ventral striatum, a connection thought to be particularly important for feeding regulation.^55^ A dopamine-dependent pathway from the ventral striatum to the hypothalamus has been described that mediates fear.^56^ This lends itself to the hypothesis that PE signal in AN might activate this circuitry and override appetitive hypothalamic signals.

Our analyses also indicate direct associations of PE response with HA, BMI change, and taste perception. Orbitofrontal cortex gyrus rectus PE was negatively correlated with BMI increase during treatment but positively correlated with HA, which in turn was positively correlated with drive for thinness and body dissatisfaction. This indicates that PE values could be directly associated with HA and BMI change in AN when in the ill state, although OFC PE was not elevated in participants with AN compared with controls. Complex behaviors are driven by the balance between neurotransmitter systems^57^ and imbalance between, for instance, dopamine and serotonin neurotransmission in those with AN could make the PE signal relatively more important in its association with HA. Our data also show that HA is directly correlated with core AN behavior, such as drive for thinness and body dissatisfaction, suggesting that anxiety is an important driver of the cognitive/emotional aspects specific to AN. This study suggests that dopamine circuits via PE signaling could be involved with elevated HA. However, the specific neurotransmitter systems underlying those results need further exploration.^9,10,12^ Pleasant taste stimulates dopamine release to promote eating and typically activates OFC response.^58,59,60^ Our data raise the possibility that adolescents with AN in this study were negatively conditioned to sweet taste and may have developed an inverse association with dopamine release across the larger reward circuitry.^61,62^ A possible explanation could be that high HA drives low taste pleasantness, making the taste experience less pleasant.

Consistent with other studies, those with AN exhibited higher AUC for cortisol levels compared with controls,^29,30^ which may alter appetite regulation in AN.^63^ Depression scores were not correlated with cortisol levels. The positive regression of AUC for cortisol level and PE signal in participants with AN suggests that stress enhances PE signals.^64^ Prediction error acts as a learning signal and affects value-driven attentional bias,^65^ and stress response may affect how individuals with AN process and form associations with salient stimuli. Although correlational, our data point to a model hypothesis for further investigation: cognitive drivers in AN, such as severe body dissatisfaction, could increase stress hormone levels, which both suppress eating and enhance PE signals.

**Figure 3.**
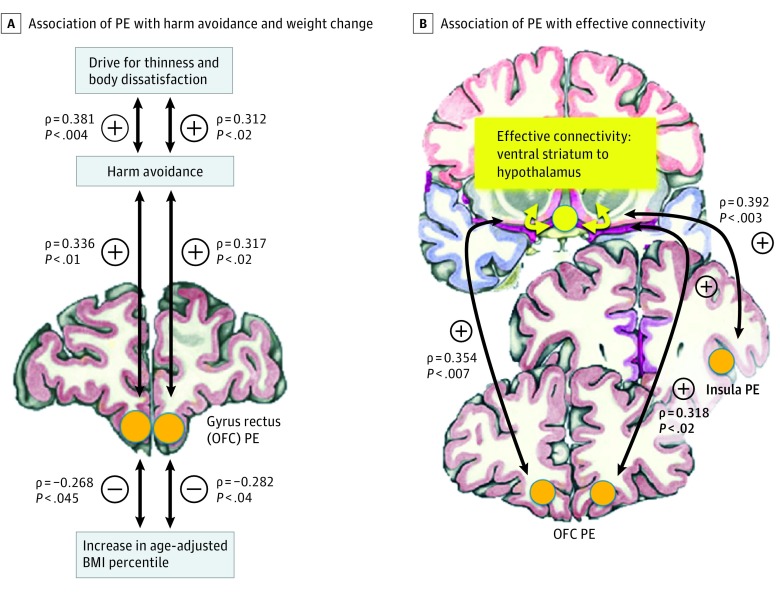
Model of Association of Reward Learning Prediction Error (PE) With Harm Avoidance, Weight Change, and Effective Connectivity in Adolescents With Anorexia Nervosa Based on the correlational results, we propose that weight loss is associated with expected sensitization of the dopamine system, reflected in elevated PE to stimulate food approach. However, increased PE may elevate anxiety (harm avoidance) in anorexia nervosa because this is in conflict with a high drive for thinness and body dissatisfaction. Prediction error activation may then become part of a fear-driven mechanism to override homeostatic signals from the hypothalamus, signals that would normally trigger eating. BMI indicates body mass index; OFC, orbitofrontal cortex.

Taking our data together with previous research, we propose the following model to explain the paradoxical food restriction in AN (Figure 3): Food restriction and weight loss are associated with sensitization of the dopamine system^14^ and reflected in AN by elevated PE signal, probably to stimulate food approach.^11,12^ However, PE response may increase HA in AN because this biological mechanism to seek out food is inconsistent with the high drive for thinness and body dissatisfaction. Thus, there is a conflict between food approach mechanisms (PE) and cognitive-emotional processes that oppose eating (body dissatisfaction and drive for thinness). Prediction error activation may then become part of a fear-driven mechanism that includes the ventral striatum to override homeostatic signals from the hypothalamus, which would normally trigger food intake. Future studies will have to test the validity of this model and test whether this circuitry indeed activates or involves the previously described fear-mediating pathway from the ventral striatum to the hypothalamus.^56^

### Limitations

This study has limitations. Functional magnetic resonance imaging does not directly measure dopaminergic signaling; the biologically based computational model used in this study provides strong evidence of altered dopamine-related taste reward processing in adolescent AN, but specific pharmacological challenge studies are needed to further support this model. The dynamic connectivity analysis included conditioned visual stimulus and unconditioned taste stimulus response; future studies will be required to separate these over the full hemodynamic response time. Power for the cortisol level analysis was limited because we were unable to collect salivary cortisol samples from the entire cohort. The analysis of 20 participants with AN and 25 controls still resulted in robust, multiple comparison–corrected findings, although replication is needed. The data were collected on 2 separate MRI scanners. However, comparison results did not indicate within-group differences. We did not assess learning rates; however, a subset of the study participants showed similar learning rates across groups in a monetary PE paradigm.^22^ Structural brain alterations could affect brain function, but there were no differences across groups (eFigure 4 in the Supplement). The age range up to 21 years was within child range by National Institute of Mental Health standards at the start of the study. However, there is no evidence that extending the current definition by 3 years would confound the results.

## Conclusions

Prediction error brain response may have a central role in adolescent AN and illness behaviors. However, longitudinal studies and neurotransmitter challenge studies are needed to further understand how brain circuits are disrupted or altered in AN. This will help to identify neurobiological systems that are involved in AN pathophysiology and to develop targeted biological interventions. Another goal will be to identify demographic, behavioral, and biological variables that can predict PE signal and can be measured without a brain scan to make this mechanism clinically more accessible and useful.

## Notes

Supplement. **eAppendix 1.** Taste reward task paradigm. **eAppendix 2.** Cortisol-brain activation regression. **eMethods 1.** 3T GE Signa and Siemens Skyra 3T scanners. **eMethods 2.** Temporal difference learning algorithm. **eMethods 3.** Effective connectivity analysis methods. **eTable 1.** Cortisol sample demographics. **eTable 2.** Taste pleasantness 1 molar sucrose solution and ROI PE response correlations. **eFigure 1.** PE analysis by scanner. **eFigure 2.** Behavioral correlations. **eFigure 3.** Cortisol analysis. **eFigure 4.** Brain volume measures.
