# Supplementary material for: Association of Brain Reward Learning Response With Harm Avoidance, Weight Gain, and Hypothalamic Effective Connectivity in Adolescent Anorexia Nervosa
Source: JAMA Psychiatry. 2018 Jul 19;75(10):1071–80. doi: 10.1001/jamapsychiatry.2018.2151 (PMC6233809; doi:10.1001/jamapsychiatry.2018.2151)
Supplement: Supplement. — eAppendix 1. Taste reward task paradigm. eAppendix 2. Cortisol-brain activation regression. eMethods 1. 3T GE Signa and Siemens Skyra 3T scanners. eMethods 2. Temporal difference learning algorithm. eMethods 3. Effective connectivity analysis methods. eTable 1. Cortisol sample demographics. eTable 2. Taste pleasantness 1 molar sucrose solution and ROI PE response correlations. eFigure 1. PE analysis by scanner. eFigure 2. Behavioral correlations. eFigure 3. Cortisol analysis. eFigure 4. Brain volume measures. [file jamapsychiatry-75-1071-s001.pdf]

Supplementary Online Content

Frank GKW, DeGuzman MC, Shott ME, Laudenslager ML, Rossi B, Pryor T. Association of brain reward learning response with harm avoidance, weight gain, and hypothalamic effective connectivity in adolescent anorexia nervosa. *JAMA Psychiatry*. Published online July 19, 2018. doi:10.1001/jamapsychiatry.2018.2151

eAppendix 1. Taste reward task paradigm
eAppendix 2. Cortisol-brain activation regression
eMethods 1. 3T GE Signa and Siemens Skyra 3T scanners
eMethods 2. Temporal difference learning algorithm
eMethods 3. Effective connectivity analysis methods
eTable 1. Cortisol sample demographics
eTable 2. Taste pleasantness 1 molar sucrose solution and ROI PE response correlations
eFigure 1. PE analysis by scanner
eFigure 2. Behavioral correlations
eFigure 3. Cortisol analysis
eFigure 4. Brain volume measures
eReferences.

This supplementary material has been provided by the authors to give readers additional information about their work.

© 2018 Frank GKW et al. *JAMA Psychiatry*.

eAppendix 1. Taste reward task paradigm

The taste reward task design was adapted from O'Doherty et al.[1] Participants received three taste stimuli during fMRI imaging (28 min. total task duration): 1 molar sucrose solution (100 trials), no solution (100 trials) and artificial saliva (80 trials). Participants learned to associate each unconditioned taste stimulus (US) with a paired conditioned visual stimulus (CS) that is probabilistically associated with its US: the CS shape for sucrose was followed in 80% of trials by sucrose solution (the other 20% were followed by no solution), and the CS shape associated with no-solution was followed in 80% of the trials by no solution (the other 20% were followed by sucrose); the CS shape for artificial saliva was always followed by saliva receipt. For each subject, the first 10 trials were fixed CS shape for sucrose followed by the delivery of US sucrose to establish an initial stable association between the CS sucrose shape and US sucrose taste.[1] All other trials were fully randomized without predetermined order. The taste stimuli were applied using a customized-programmable syringe pump (J-Kem Scientific, St Louis, MO, USA) controlled with the E-Prime Software (Psychological Software Tools, Pittsburgh, PA, USA). The MRI scanner radiofrequency pulse triggered taste application.[2]

Study participants were compensated for their study participation with a total of $160, prorated for completion of questionnaires, diagnostic assessment and brain imaging scan.

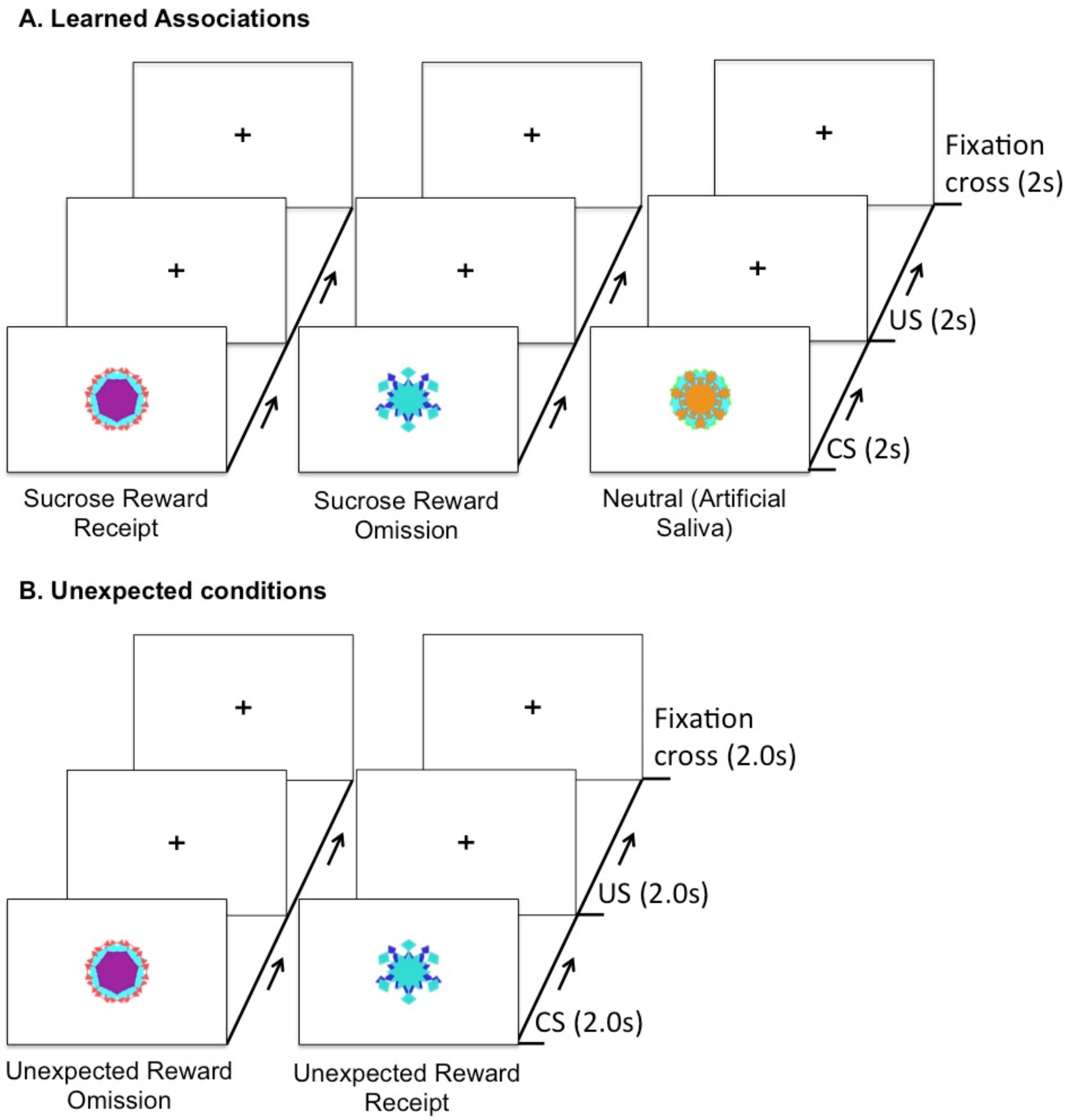

Panel A depicts the learned associations between the conditioned stimulus (CS, colored geometric shape, presented for 2 seconds (s)) and the unconditioned stimulus (US, 1ml sweet taste reward, presented for 2s). Intertrial interval was 6s. Panel B depicts the unexpected conditions where learned associations were violated during 20% of the trials.

© 2018 Frank GKW et al. *JAMA Psychiatry*.

eAppendix 2. Cortisol-brain activation regression

On the scan day, a subset of the AN (n=20) and HC (n=25) participants provided 0.5 ml of saliva samples in 5 ml sterile plastic o-ring sealed tubes by unstimulated passive drool (see supplement). Samples were collected 30 minutes prior to breakfast, 30 minutes after completed breakfast, and right before starting the scan. Samples were stored at -15° C until analysis. Cortisol was assayed using commercial immunoassays (Salimetrics, State College, PA). The area under the curve (trapezoid method, three time points) was calculated and correlated across the whole brain using SPM12 at $p_{FWE}<0.05$, k=0 including covariates for age, antipsychotic and antidepressant use, and comorbid mood and anxiety disorders.

Cortisol AUC significantly positively correlated with PE response in the right superior frontal gyrus (x=-18, y=58, z=6) in the AN group (peak $p_{FWE}<0.005$, k=1)

cluster				peak							
p FWE-corr	p FDR-corr	k_E	p unc	p FWE-corr	p FDR-corr	T	Z_E	p unc	x {mm}	y {mm}	z {mm}
0.0152	0.2285	1	0.2285	0.0047	0.1702	11.0178	5.4255	2.89E-08	-18	58	6

Subsequent small volume correction ($p<0.001$, k=10) within the anatomical superior frontal gyrus ROI resulted in a significant cluster (peak $p_{FWE}<0.001$, k=53).

cluster				peak							
p FWE-corr	p FDR-corr	k_E	p unc	p FWE-corr	p FDR-corr	T	Z_E	p unc	x{mm}	y{mm}	z{mm}
0.0147	0.2280	53	0.0228	0.0001	0.0060	11.0178	5.4255	0.0001	-18	58	6
				0.0723	0.9156	5.4838	3.8788	0.0001	-24	62	16
				0.4070	0.9156	4.0147	3.1805	0.0007	-16	60	22
0.0477	0.3761	30	0.0752	0.2426	0.9156	4.4853	3.4256	0.0003	-26	2	66
				0.2805	0.9156	4.3586	3.3618	0.0004	-22	4	64
				0.3355	0.9156	4.1975	3.2784	0.0005	-20	-2	68
				0.4148	0.9156	3.9961	3.1703	0.0008	-16	2	58
0.3461	0.7649	2	0.6535	0.3466	0.9156	4.1675	3.2625	0.0006	-22	22	60
0.3918	0.7649	1	0.7649	0.4374	0.9156	3.9433	3.1413	0.0008	-24	44	20

© 2018 Frank GKW et al. *JAMA Psychiatry*.

eMethods 1. 3T GE Signa and Siemens Skyra 3T scanners

Brain imaging was performed between 0700 and 0900 hours on a Siemens Skyra 3T scanner for 17 HC and 14 AN with a three-plane scout scan (16 seconds), coronally acquired, spoiled gradient sequence T1-weighted (192 slices, thickness=1.2 mm, TI=900 ms, TR=2.3 ms, TE=2.24 ms, flip angle=8°, FOV=22 cm, scan matrix=256x256), and T2*-weighted echo planar imaging scans for blood-oxygen-level-dependent (BOLD) functional activity during task performance (3.4×3.4×2.6 mm voxels, TR=2.1 seconds, TE=30 ms, flip angle=70°, 34 axial slices, thickness=2.6 mm, gap=1.4 mm). A scanner covariate was therefore included in the MANCOVA for imaging group contrasts. For prediction error results (Figure 1), the main findings still held when excluding the scanner covariate but keeping the other covariates, although with slightly higher p-values (R caudate head, $p<.008$; L caudate head, $p<0.001$; R ventral anterior insula, $p<0.005$; R nucleus accumbens, $p<0.030$; L nucleus accumbens, $p<0.010$).

© 2018 Frank GKW et al. *JAMA Psychiatry*.

eMethods 2. Temporal difference learning algorithm

The predicted value ($\hat{V}$) at any time (t) within a trial is calculated as a linear product of weights (w_i) and the presence of a conditioned visual stimulus (CS) at time t, coded in a stimulus representation vector $x_i(t)$ where each stimulus x_i is represented separately at each moment in time:

$$V(t) = \sum_i W_i x_i(t)$$

Predicted stimulus value at time t is updated by comparing the predicted value at time t+1 to that actually observed at time t, leading to the prediction error $\delta(t)$:

$$\delta(t) = r(t) + \gamma\hat{V}(t+1) - \hat{V}(t)$$

where $r(t)$ is the reward at time t. The parameter γ is a discount factor, which determines the extent to which rewards arriving sooner are more important than rewards that arrive later during the task, with γ=0.99. The weights wi relate to how likely a particular unconditioned reward stimulus (US) follows the associated CS and are updated on each trial according to the correlation between prediction error and the stimulus representation:

$$\Delta w_i = \alpha \sum_t x_i(t)\delta(t)$$

where α is a learning rate. Between slow and fast learning rates, (0.2, 0.7) a slow α=0.2 was the best fit for study groups. Initial reward values were 1 for Sucrose Receipt and 0 for No Sucrose. Trial-to-trial prediction error was regressed with brain activation across all trials within each subject.

The prediction error calculated for each trial was modeled as an absolute (reflecting response strength) without separating positive or negative prediction error trials. This trial-to-trial calculated prediction error was then regressed with the parameter estimates derived from brain

© 2018 Frank GKW et al. *JAMA Psychiatry*.

activation across all trials within each subject. Parameter estimates were then extracted for further analysis.

© 2018 Frank GKW et al. *JAMA Psychiatry*.

eMethods 3. Effective connectivity analysis methods

We used the SPM12 MarsBar toolbox to exctract functional time-series data for the previously examined[3] expected receipt of 1 M sucrose solution. The TETRAD V program[4] was next used to infer effective connectivity with Independent Multiplesample Greedy Equivalence Search (IMaGES) and Linear non-gaussian Orientation, Fixed Structure search algorithms. This analysis aimed to understand causal relations among neuronal populations whose activity gives rise to observed fMRI signals in spatially localized regions of interest. For detailed methods, Results analyses are presented as directed graphs, where nodes or vertices in the graph represent brain regions and directed edges in the graph represent relatively direct causal influences of one region on another. The Independent Multiplesample Greedy Equivalence Search (IMaGES) is a modification of the Greedy Equivalence Search (GES) that allows analysis of multiple data sets. GES begins with an empty graph whose vertices are the recorded variables and proceeds to search forward, one new connection at a time, over Markov Equivalence classes of directed acyclic graphs. Each class of models with an additional edge is scored using the Bayes Information Criterion: $-2\ln(ML) + k \ln(n)$, where ML is the maximum likelihood estimate, k is the dimension of the model (the number of directed edges plus the number of variables) and n is the sample size. The algorithm searches forward from the empty graph until no improvement in the Bayes Information Criterion score is possible, and then backward, and outputs a description of a Markov Equivalence class. The algorithm requires a computation of a series of maximum likelihood estimates and is limited to cases where approximations to such estimates can be rapidly obtained. The analysis process in IMaGES and GES is nonlinear, and therefore a comparison of a parameterized output of the GES using conventional linear models for group comparison is not recommended. IMaGES was supplemented by a Linear non-gaussian

© 2018 Frank GKW et al. *JAMA Psychiatry*.

Orientation, Fixed Structure algorithm postprocessor; this leads to a precision of orientations that is greater than 90% and the precision of recall greater than 80%, that is, more edges are directed than with IMaGES alone, and with no loss of accuracy.[5]

© 2018 Frank GKW et al. *JAMA Psychiatry*.

eTable 1. Cortisol sample demographics[a]

Population		Min	Max	Mean	SD
HC	Age Scan (Years)	12.08	21.58	15.86	2.96
	Age Adjusted Body Mass Index Percentile (BMI)	29.50	93.40	58.62	18.94
	BMI	18.15	25.13	20.74	2.23
	Harm avoidance	4	19	10.64	3.89
	Drive for thinness	0	11	2.08	2.98
	Body dissatisfaction	0	11	3.16	3.62
	Sensitivity to Punishment	2	14	5.88	3.46
	State anxiety	20	41	28.72	6.30
	Trait anxiety	20	50	30.24	7.36
AN	Age Scan (Years)	11.92	20.71	16.33	2.21
	Age Adjusted Body Mass Index Percentile (BMI)	0.10	9.50	2.82	2.69
	BMI	14.59	17.56	16.04	0.88
	Harm avoidance	4	33	19.80	7.72
	Drive for thinness	4	28	19.20	7.19
	Body dissatisfaction	5	40	23.25	11.66
	Sensitivity to Punishment	6	18	11.90	3.81
	State anxiety	24	71	51.11	13.98
	Trait anxiety	24	77	52.75	12.84

[a]Independent t-tests showed no significant differences in any of the above measures between the subgroups and the larger sample included in the PE analysis.

© 2018 Frank GKW et al. *JAMA Psychiatry*.

eTable 2. Taste pleasantness 1 molar sucrose solution and ROI PE response correlations

**Ranked across group, PEARSON**	**HC**							**AN**							**HC vs AN**			
	**correlation**	**sig. (2-tailed)**		**bootstrap (1000 samples)**		**95% CI**		**correlation**	**sig. (2-tailed)**		**bootstrap (1000 samples)**		**95% CI**		**Fisher's z**	**p-value**	**zou's CI**	
**Pleasantness with:**	**r**	**p**	**R2**	**bias**	**SE**	**Lower**	**Upper**	**r**	**p**	**R2**	**bias**	**SE**	**Lower**	**Upper**				
R caudate head	-0.111	0.435	0.012	-0.001	0.132	-0.369	0.150	-0.418	0.001	0.174	0.005	0.108	-0.616	-0.190	1.683	0.092	-0.051	0.647
**L caudate head**	**-0.041**	**0.774**	**0.002**	**0.000**	**0.145**	**-0.334**	**0.251**	**-0.428**	**<0.0009**	**0.183**	**0.004**	**0.098**	**-0.620**	**-0.227**	**2.103**	**0.036**	**0.0252**	**0.724**
R inferior orbitofrontal cortex	-0.067	0.636	0.005	-0.002	0.149	-0.370	0.225	-0.336	0.011	0.113	0.007	0.114	-0.543	-0.091	1.424	0.154	-0.101	0.619
L inferior orbitofrontal cortex	-0.152	0.283	0.023	0.002	0.142	-0.417	0.131	-0.373	0.005	0.139	0.007	0.114	-0.575	-0.132	1.206	0.228	-0.1372	0.568
R medial orbitofrontal cortex	0.040	0.780	0.002	-0.009	0.150	-0.263	0.316	-0.249	0.064	0.062	0.006	0.120	-0.488	-0.002	1.483	0.138	-0.093	0.644
**L medial orbitofrontal cortex**	**-0.026**	**0.852**	**0.001**	**-0.005**	**0.147**	**-0.322**	**0.246**	**-0.433**	**<0.0006**	**0.187**	**0.006**	**0.110**	**-0.640**	**-0.212**	**2.204**	**0.028**	**0.044**	**0.741**
R middle orbitofrontal cortex	-0.328	0.017	0.108	-0.004	0.123	-0.563	-0.091	-0.400	0.002	0.160	0.006	0.109	-0.598	-0.157	0.418	0.676	-0.261	0.406
L middle orbitofrontal cortex	-0.123	0.386	0.015	0.004	0.134	-0.384	0.154	-0.290	0.030	0.084	0.007	0.115	-0.501	-0.058	0.885	0.376	-0.201	0.524
R dorsal anterior insula	-0.083	0.560	0.007	0.002	0.155	-0.390	0.241	-0.267	0.047	0.071	0.007	0.115	-0.478	-0.031	0.964	0.335	-0.189	0.544
L dorsal anterior insula	-0.128	0.365	0.016	0.003	0.159	-0.444	0.196	-0.296	0.027	0.088	0.008	0.118	-0.505	-0.057	0.890	0.374	-0.199	0.524
R ventral anterior insula	-0.002	0.991	0.000	0.003	0.155	-0.310	0.312	-0.272	0.042	0.074	0.005	0.116	-0.492	-0.032	1.402	0.161	-0.108	0.626
L ventral anterior insula	-0.051	0.717	0.003	0.005	0.158	-0.361	0.270	-0.338	0.011	0.114	0.008	0.114	-0.549	-0.105	1.515	0.130	-0.084	0.636

© 2018 Frank GKW et al. *JAMA Psychiatry*.

**R ventral caudate/nucleus accumbens**	**-0.098**	**0.490**	**0.010**	**0.001**	**0.131**	**-0.347**	**0.176**	**-0.451**	**<0.00009**	**0.204**	**0.004**	**0.103**	**-0.643**	**-0.245**	**1.958**	**0.050**	**-0.001**	**0.688**
**L ventral caudate/nucleus accumbens**	**0.009**	**0.949**	**0.000**	**-0.001**	**0.135**	**-0.257**	**0.284**	**-0.418**	**<0.0007**	**0.175**	**0.003**	**0.109**	**-0.624**	**-0.193**	**2.293**	**0.022**	**0.061**	**0.762**
R gyrus rectus	-0.007	0.961	0.000	-0.004	0.145	-0.317	0.279	-0.333	0.012	0.111	0.004	0.102	-0.524	-0.110	1.712	0.087	-0.048	0.674
L gyrus rectus	-0.052	0.716	0.003	-0.004	0.144	-0.335	0.237	-0.360	0.006	0.130	0.005	0.119	-0.593	-0.110	1.641	0.101	-0.060	0.655

© 2018 Frank GKW et al. *JAMA Psychiatry*.

eFigure 1. PE analysis by scanner[a]

The left column in each group (HC, healthy controls; AN, adolescents with anorexia nervosa) shows data from the 3T GE Signa scanner and the right column from the Siemens Skyra 3T scanner.

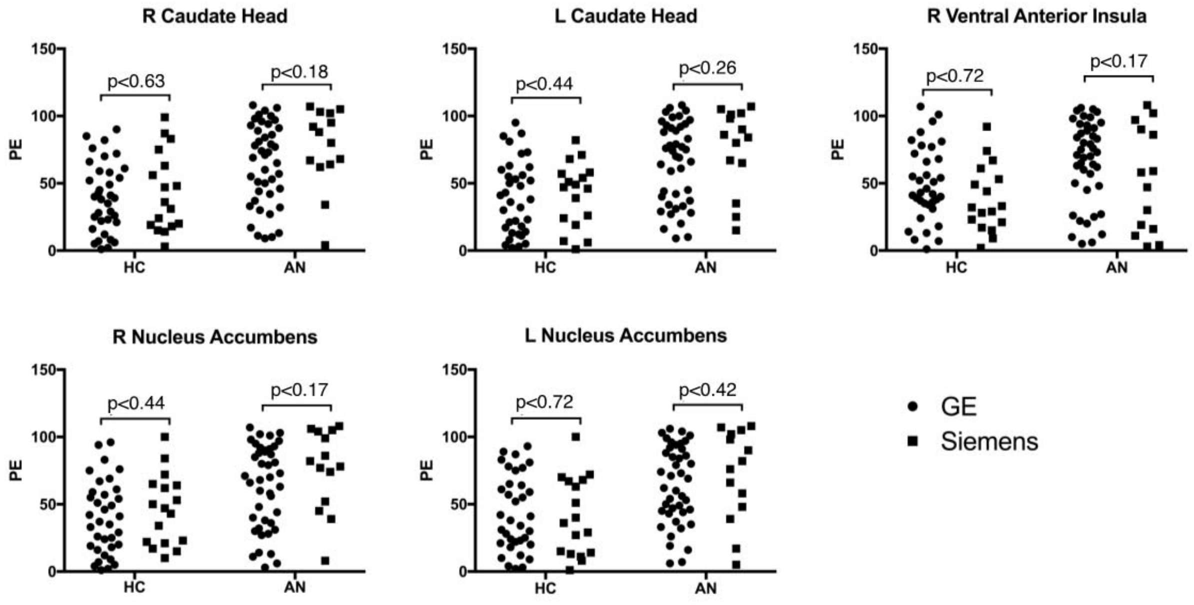

[a]Independent t-tests within group of ranked PE showed no significant differences between scanners (p>0.05).

© 2018 Frank GKW et al. *JAMA Psychiatry*.

eFigure 2. Behavioral correlations

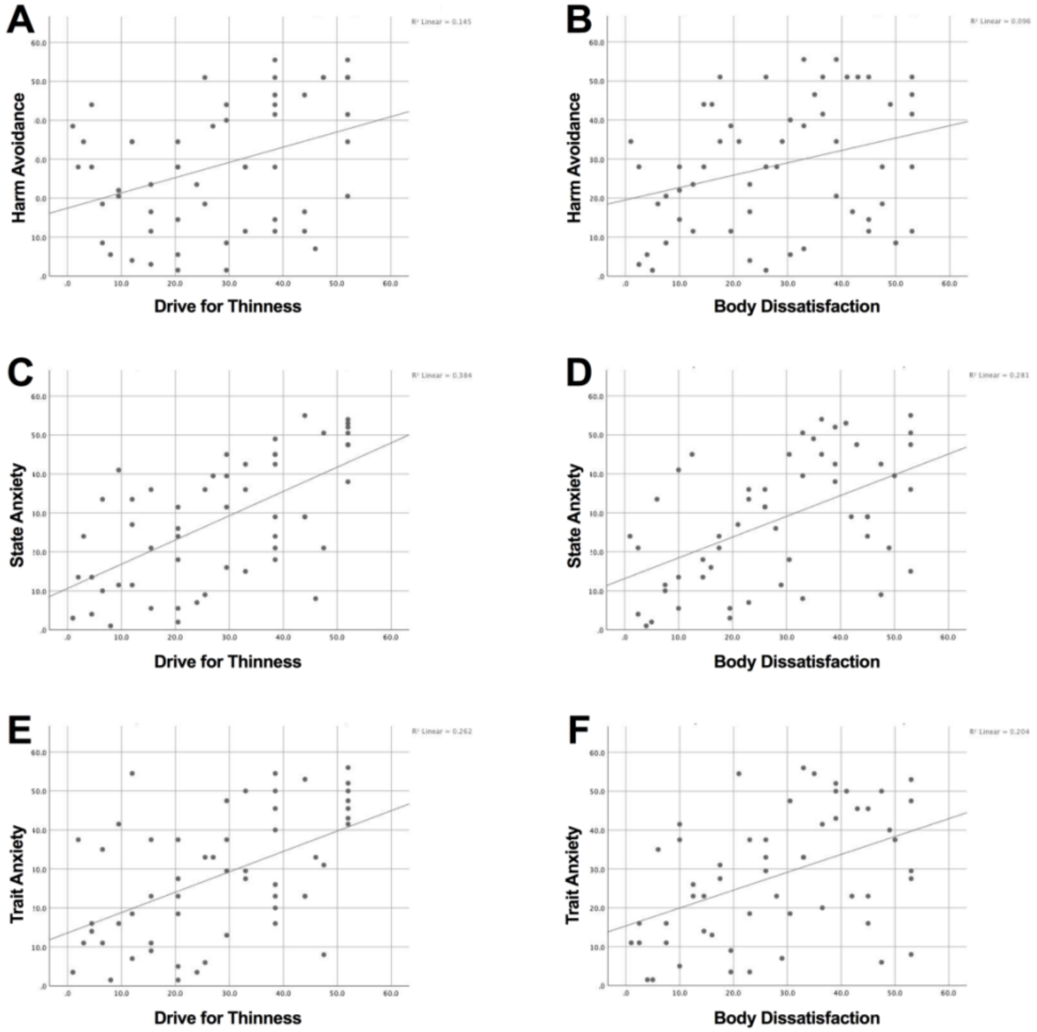

Drive for thinness correlates positively with (A) Harm avoidance, $p<0.004$, $R^2=0.145$, (C) State anxiety, $p<0.001$, $R^2=0.384$, and (E) Trait anxiety, $p<0.001$, $R^2=0.262$. Body dissatisfaction positively correlated with (B) Harm avoidance, $p<0.02$, $R^2=0.096$, (D) State anxiety, $p<0.001$, $R^2=0.281$, (F) Trait anxiety, $p<0.001$, $R^2=0.262$. All p-values are corrected for multiple comparisons (FDR).

© 2018 Frank GKW et al. *JAMA Psychiatry*.

eFigure 3. Cortisol analysis

A

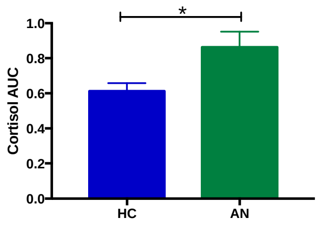

B

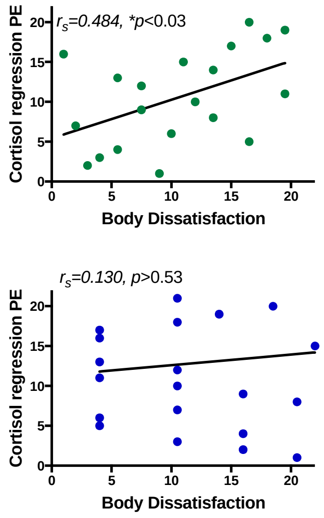

A. Cortisol levels were significantly higher in AN (green bar) than HC (blue bar), *p<0.5. B. Parameter estimates (PE) extracted from the FWE corrected cluster positively correlated with body dissatisfaction in the AN (top panel) but not in the HC group (bottom panel).

© 2018 Frank GKW et al. *JAMA Psychiatry*.

eFigure 4. Brain volume measures[a]

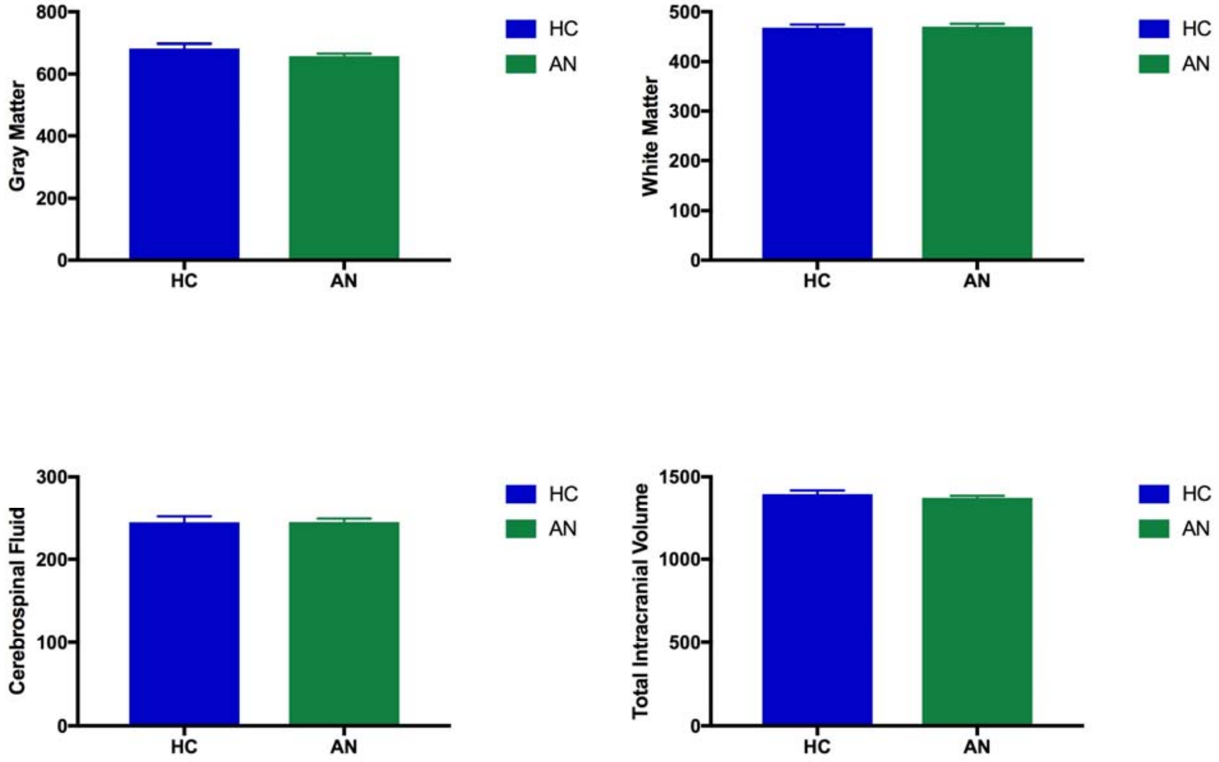

[a]Bar graphs depict brain volume differences between groups were not significant for whole brain gray matter, white matter, cerebrospinal fluid, or total intracranial volume. Healthy control means (N=52) are indicated by blue bars, anorexia nervosa group means (N=56) are indicated by green bars. Error bars = +/- 1 standard error of the mean.

© 2018 Frank GKW et al. *JAMA Psychiatry*.

eReferences.

1. O'Doherty JP, Dayan P, Friston K, Critchley H, Dolan RJ. Temporal difference models and reward-related learning in the human brain. *Neuron.* 2003;38(2):329-337.

2. Frank GK, Reynolds JR, Shott ME, O'Reilly RC. Altered temporal difference learning in bulimia nervosa. *Biological Psychiatry.* 2011;70(8):728-735.

3. Frank GK, Shott ME, Riederer J, Pryor T. Altered Structural and Effective Connectivity in Anorexia and Bulimia Nervosa in Circuits that Regulate Energy and Reward Homeostasis. *Translational Psychiatry*. 2016;6(11):e932.

4. Ramsey JD, Hanson SJ, Hanson C, Halchenko YO, Poldrack RA, Glymour C. Six problems for causal inference from fMRI. *NeuroImage.* 2010;49(2):1545-1558.

5. Ramsey JD, Hanson SJ, Glymour C. Multi-subject search correctly identifies causal connections and most causal directions in the DCM models of the Smith et al. simulation study. *NeuroImage.* 2011;58(3):838-848.

© 2018 Frank GKW et al. *JAMA Psychiatry*.
